# Biologically-Directed Modeling Reflects Cytolytic Clearance of SIV-Infected Cells *In Vivo* in Macaques

**DOI:** 10.1371/journal.pone.0044778

**Published:** 2012-09-13

**Authors:** W. David Wick, Otto O. Yang

**Affiliations:** 1 Vaccine and Infectious Disease Division, Fred Hutchinson Cancer Research Center, Seattle, Washington, United States of America; 2 Geffen School of Medicine, Department of Medicine/Department of Microbiology, Immunology, and Molecular Genetics/UCLA AIDS Institute, University of California Los Angeles, Los Angeles, California, United States of America; University of Pittsburgh Center for Vaccine Research, United States of America

## Abstract

The disappointing outcomes of cellular immune-based vaccines against HIV-1 despite strong evidence for the protective role of CD8^+^ T lymphocytes (CTLs) has prompted revisiting the mechanisms of cellular immunity. Prior data from experiments examining the kinetics of Simian Immunodeficiency Virus (SIV) clearance in infected macaques with or without *in vivo* CD8 depletion were interpreted as refuting the concept that CTLs suppress SIV/HIV by direct killing of infected cells. Here we briefly review the biological evidence for CTL cytolytic activity in viral infections, and utilize biologically-directed modeling to assess the possibility of a killing mechanism for the antiviral effect of CTLs, taking into account the generation, proliferation, and survival of activated CD4^+^ and CD8^+^ T lymphocytes, as well as the life cycle of the virus. Our analyses of the published macaque data using these models support a killing mechanism, when one considers T lymphocyte and HIV-1 lifecycles, and factors such as the eclipse period before release of virions by infected cells, an exponential pattern of virion production by infected cells, and a variable lifespan for acutely infected cells. We conclude that for SIV/HIV pathogenesis, CTLs deserve their reputation as being cytolytic.

## Introduction

Clinical failure of a promising T-cell based HIV-1 vaccine in a phase IIb human trial (STEP) [1] has prompted a re-evaluation of the mechanisms of immunity, because tests of T-cell based vaccines against SIV in macaques have indicated that virus-specific CD8^+^ T lymphocytes (CTLs) can ameliorate or even prevent infection. Several macaque studies of recombinant adenovirus-based vaccines similar to the one tested in STEP have demonstrated prevention or control of chronic viremia after SIV challenge in the absence of protective antibody responses (by lack of envelope inclusion in the vaccine and/or lack of neutralizing antibody responses) [2], [3], [4]. These results indicate the possibility that vaccine-elicited CTLs might provide chronic suppression of symptomatic infection, or even abort early retroviral infection as predicted by a mathematical model [5].

That CTLs clear acute viral infections or control chronic viral infections is well established, and they play a protective albeit ultimately unsuccessful role in HIV/SIV pathogenesis. For HIV-1, the rapid evolution of immune-targeted sequences [6], [7], [8] and temporal association of developing CTL responses to the drop of peak viremia ending acute infection [9], [10] provide strong evidence for immune pressure by CTLs. Perhaps the most direct evidence comes from experiments in which CD8^+^ cells in SIV-infected macaques are depleted with an anti-CD8 monoclonal antibody *in vivo*, which results in a massive concomitant rise in viremia [11], [12], [13], [14].

There are two major proposed mechanisms whereby CTLs exert antiviral pressure. The first study demonstrating CTL antiviral activity against HIV-1 [15] and further work by the same investigators led to the conclusion that a noncytolytic soluble factor is responsible [16]. It was proposed that CTLs release this factor through a non-MHC-restricted, and therefore non-epitope-specific manner [17]. Subsequent observations of viral evolution in response to CTLs *in vivo* [18], [19], [20] have made it clear that the major antiviral activity of CTLs is MHC-I-dependent and therefore epitope-specific, although this does not exclude the possibility that the effector mechanism is mediated by non-cytolytic factor(s) in part or in whole, as shown by release of anti-HIV-1 soluble factors in an epitope-dependent manner [21], [22]. The other major proposed mechanism is direct cytolysis by MHC class I-restricted, epitope-specific CTLs. Several observations strongly favor this mechanism. CTLs from HIV-1-infected persons can kill HIV-1 protein-expressing target cells directly *ex vivo* [23], [24]. CTL clones *in vitro* can kill HIV-1-infected cells [25] and suppress HIV-1 replication predominately through direct cytolysis [22]. Preservation of CTL expression of perforin and granzyme correlates to effective immune control of HIV-1 infection *in vivo* [26]. Murine antiviral CTLs can be demonstrated to kill target cells *in vivo* [27]. Finally, mathematical models reproducing the dynamics of acute and chronic HIV-1 infection provide compatible estimates of *in vivo* and *in vitro* CTL killing of HIV-1-infected cells [28], [29].

However, two reports in PLoS Pathogens [30], [31] have presented experiments with SIV-infected macaques, examining dynamics of viremia after administration of antiretroviral therapy (ART) in the presence or absence of monoclonal antibody-mediated CD8 depletion *in vivo*. Comparing small numbers of macaques with and without depletion (three to five per group), these studies could not find a significant difference in the rates of viremia decay, and both concluded using a mathematical analysis of viral decay rate [32] (“fixed production model”) that the lifespan of infected cells was not detectably lengthened by CD8 depletion and thus the antiviral effect of CTLs could not involve cytolysis. Both studies suggested that the lifespan of an SIV-infected CD4^+^ T lymphocyte is about one to two days, and Wong *et al* [31] further stated that for loss of infected cell cytolysis by CTLs to mediate the rise in viremia seen after CD8 depletion, it “would require … a 10-fold increase in productive cell lifespan for the mathematical model used here,” which was not observed. It therefore was concluded in both studies that CTLs exert antiviral effects entirely through a non-cytolytic mechanism.

However, these predictions are inconsistent with several biological properties of T lymphocytes and SIV infection. Acutely infected CD4^+^ T lymphocytes have a significant “eclipse period” of one to two days before virion release [25], [33]; this is incompatible with the total infected cell lifetime of one to two days predicted by application of the fixed production model in these two studies (which allotted no eclipse period). Additionally, virion production during the productive phase of cell infection is exponential [25], [33] and not linear as assumed implicitly in that model. Furthermore, the major target cell for viral infection, the activated effector-memory CD4^+^ T lymphocyte, has a short lifespan of days after differentiating from the long-lived central memory pool of CD4^+^ T lymphocytes [34], [35], and it is inconceivable that an infected effector-memory CD4^+^ T lymphocyte could survive the 10 to 20 days required to demonstrate cytolysis as predicted by the fixed production model. Here, we propose that more detailed models that consider the biology of virus and T lymphocyte dynamics do in fact support CTL cytolytic activity as an important antiviral mechanism in the immunopathogenesis of SIV/HIV infection.

## Methods

### Modeling CD4^+^ T Lymphocyte Turnover and Infection by SIV: Non-programmed Proliferation (NPP) Model

**Figure 1:**
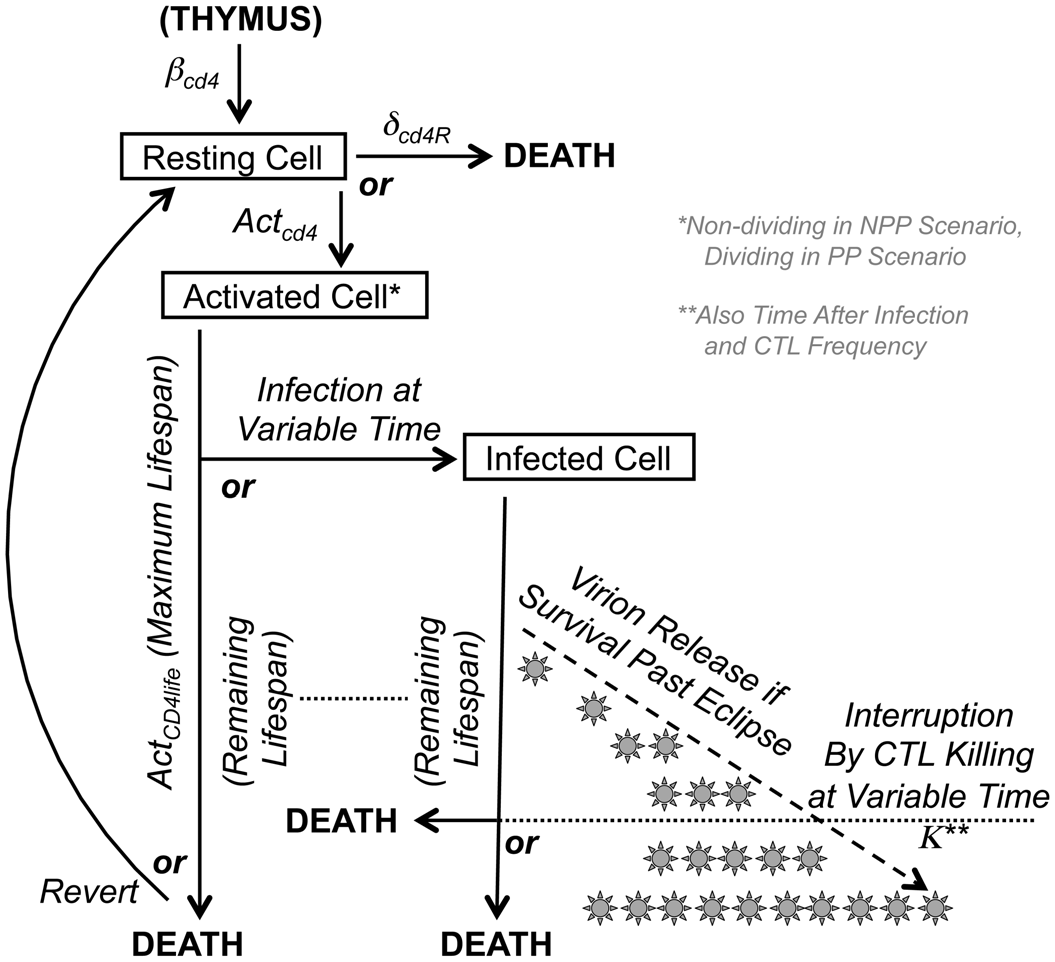
Schematic of the CD4^+^ T lymphocyte and SIV replication model. The modeling process for generation, activation, infection, and death of the CD4^+^ T lymphocyte compartment is displayed schematically, with relevant biological parameters indicated.

Figure 1 outlines the general design of this simulation, and mathematical details are given in the Supplemental Methods. The CD4^+^ T lymphocyte compartment is modeled in simple terms of production, activation, and death, because these are the relevant parameters determining viral replication and target cell availability. Because activated memory cells are overwhelmingly the major source of SIV replication, we designated CD4^+^ T lymphocytes only as resting or activated, without further distinguishing resting cells as naïve or memory because neither support significant viral replication. It is assumed that resting CD4^+^ T lymphocytes are produced and activated at a constant rate without proliferation (non-programmed proliferation, NPP), in which case some cells return to resting and other cells die and are replaced by new resting cells (through thymic output or homeostatic proliferation of resting cells). The computer simulation considers the life of a cell in terms of small incremental time units (“stages”) during which the cell can survive or die, change activation state (activated versus resting), and become infected if activated. Uninfected activated cells can survive for a set lifespan before either dying or reverting to a resting state, and become infected at any time after activation. An infected cell can only survive up to the maximum time the cell would have survived if uninfected. Once infected, viral replication follows a set timing of an intracellular phase (“eclipse” before any virus is released) followed by exponentially increasing virion release for the remainder of the cell’s life. An activated cell infected at an early stage of life may survive to produce a fixed maximum number of virions, or die before that level is reached. Furthermore, as described in the next section, with increasing time after infection, the cell is susceptible to killing by a CTL before the maximum is reached, with a killing rate that is directly proportional to the phase of viral replication. Newly produced virions infect other activated CD4^+^ T lymphocytes or are rapidly cleared from circulation. The biologic constants utilized in this model are listed in Table 1.

**Table 1:** Biologic parameters for modeling of the CD4^+^ T lymphocyte compartment.

Parameter	Definition	Value
*N_CD4_*	Total body CD4^+^ T lymphocytes at steady state (before SIV infection)	3.5×10^9^ cells [44]
*δ_CD4R_*	Death rate of resting CD4^+^ T lymphocytes	3×10^−3^/day [45]
*ActCD4fract*	Fraction of activated CD4^+^ T lymphocytes at steady-state before SIV infection	4.5×10^−2^ [44], [46]
*Act_CD4_*	Constant rate of activation of naïve CD4^+^ T lymphocytes	1.2×10^−2^/day*
*β_CD4_*	Rate of new naïve CD4^+^ T lymphocyte production	4.75×10^7^ cells/day**
*ActCD4life*	Lifespan of an activated CD4^+^ T lymphocyte	4 days [47]
*S*	Number of temporal subdivisions of the lifespan utilized for the simulation	50
*Revert*	Reversion rate of activated CD4^+^ T lymphocytes returning to resting	5×10^−2^ [36], [51]
*I_Ini_*	Initial number of SIV-infected activated CD4^+^ T lymphocytes	Set at 100 cells
*Eclipse*	Time before an infected CD4^+^ T lymphocyte starts to release virions	2 days [25], [33]
*Virion Prod*	Maximum virus that an infected CD4^+^ T lymphocyte can produce	2×10^4^ virions/day [48], [64]
*R_0_*	Basic reproductive number (number of new infected cells from one productivelyinfected cell in absence of immune response)	10 cells***
*κ*	Factor for CTL killing of infected cells relative to stage of cell infection	5×10^−9^/cell/day [29]
*δ_V_*	Lifespan of a free virion	0.5 hour [60], [61]

The parameters utilize for modeling CD4^+^ T lymphocyte turnover and infection using the NPP assumption are listed. For the PP assumption, activated cells were committed to 8 divisions with the same kinetics as CTLs (see Table 2).

* Derived constant based on biologically observed steady state value and estimated loss rate of activated cells (see Supplemental Methods). For the programmed proliferation assumption (PP), the value is 1.9×10^−4^/day.

** Derived constant based on biologically observed steady state value and estimated loss rate of total body CD4^+^ T lymphocytes (see Supplemental Methods). For the programmed proliferation assumption (PP), the value is 2.5×10^6^/day.

*** Set constant based on the model yielding timing of peak viremia corresponding to biologically observed timing, and consistent with virion viability ranging from 1∶1 to 1∶1000 [64] and *Virion Prod*.

### Modeling CD4^+^ T Lymphocyte Turnover and Infection by SIV: Programmed Proliferation (PP) Model

This alternative model for production of target activated CD4^+^ T lymphocytes followed the same general structure as the NPP model (Figure 1) but employed an alternative assumption for generation of cells (similar to that used for modeling the CTL compartment, below), that upon activation the cells are committed to undergo proliferation for a fixed number of divisions [36], [37]. Once stimulated and activated, cells then undergo 8 divisions, during which they are susceptible to SIV infection in the same manner as the NPP model described above. After a cell is infected, it does not complete the remaining program of cell divisions, due to effects of infection including cell cycle arrest (mediated by viral replication and/or Vpr [38], [39]), and thus follows the same pattern as the NPP model.

### Modeling SIV-specific CD8^+^ T Lymphocytes (CTLs)

**Figure 2:**
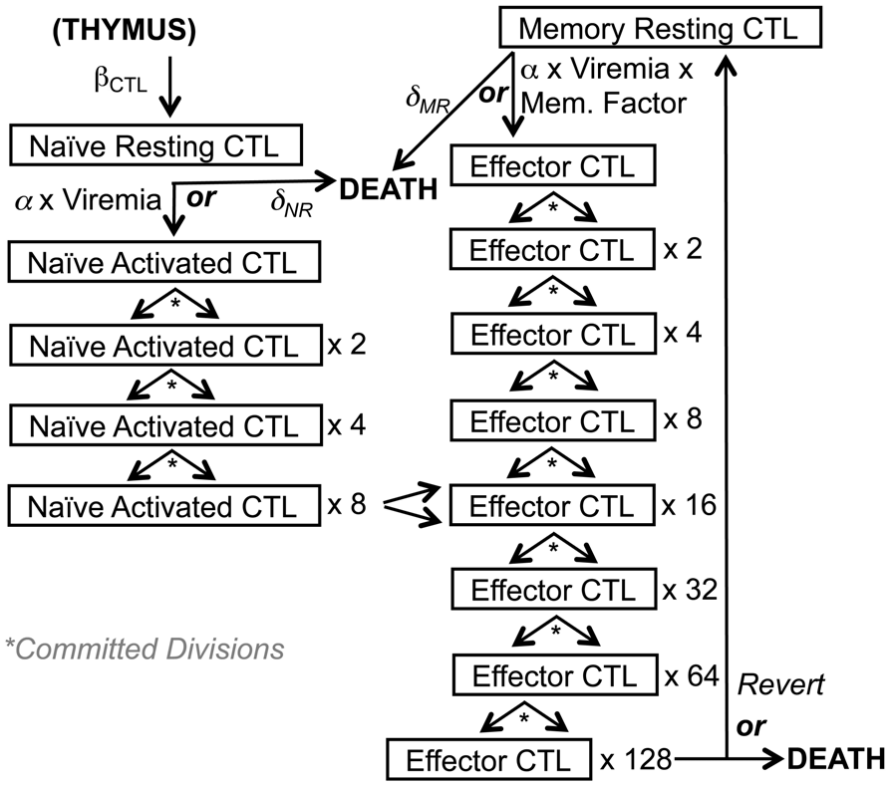
Schematic of the SIV-specific CTL model. The modeling process for generation, antigenic activation, and death of the SIV-specific CD8^+^ T lymphocyte (CTL) compartment is displayed schematically, with relevant biological parameters indicated.

The modeling of SIV-specific CTLs was a modification of one that we previously described [28], [29], [40], [41], and is outlined schematically in Figure 2. This model starts with a fixed number of naïve SIV-specific CTLs, which are activated by exposure to SIV at an activation rate dependent on viremia (which is also equivalent to the killing rate of infected cells), after which they undergo a programmed proliferation commitment of eight divisions [36], [37], differentiating into activated effector CTLs at the fourth division. These cells then either die or revert to resting memory CTLs, which are primed to respond immediately as mature effector CTLs upon re-exposure to antigen. Naïve and resting CTLs have distinct death rates. Viral escape from CTLs was not modeled, since CTLs and viral epitope sequences reach a steady state in chronic infection [42], [43]. The biologic constants utilized in this model are listed in Table 2.

**Table 2:** Biologic parameters for modeling of SIV-specific CD8^+^ T lymphocyte compartment.

Parameter	Definition	Value
*IniNR*	Initial number of naïve SIV-specific CTLs in a macaque	8.75×10^4^ [44], [49] *
*α*	Factor for naïve SIV-specific CTL activation relative to virus production	5×10^−9^/cell/day [29]
*δ_NR_*	Naïve (resting) CTL death rate	10^−3^/day [45], [46]
*β_CD8_*	Production rate of naïve SIV-specific CTLs	87.5 cells/day**
*D_CD8_*	Number of CTL divisions after antigenic stimulation	8 [36], [37]
*Revert*	Reversion rate of activated CTLs returning to resting	5×10^−2^ [36], [51]
*δ_MR_*	Memory resting CTL death rate	3×10^−2^/day [52], [53], [54]
*Memory Factor*	Ratio of memory versus naïve CTL activation rates	7 [46], [47]
*CC_naive_*	Mitosis time for naïve CTLs	12 hours [55]
*CC_memory_*	Mitosis time for memory CTLs	6 hours [55]
*NCTL_d_*	Doublings for a naïve CTL to become an effector CTL	4 doublings [55]
*MCTL_d_*	Doublings for a resting memory CTL to become an effector CTL	1 doubling [55]

The biological constants utilized for modeling of SIV-specific CTLs are listed.

* Based on Sopper *et al* describing a total body number of 3.5×10^9^ CD8^+^ T lymphocytes and Blattman *et al* demonstrating the frequency of epitope precursors to be 5×10^−6^, and assuming a persisting CTL response to 5 epitopes.

** Derived constant calculated from biologically observed steady state value for total body CTLs, frequency of epitope-specific CTLs and loss rate of epitope-specific CTLs (see Supplemental Methods).

### Modeling the Effects of Antiretroviral Treatment in the Absence or Presence of CD8 Depletion

The models were run with the assumption of immediate and fully effective interruption of viral replication (by antiretroviral treatment) at 94 days after infection, by dropping the reproductive rate to 0 and observing the rate of viral decay as the remaining infected cells produce virus until their deaths. The slope of viral decay after treatment was determined by least squares regression fit to 28 days of viremia. They were re-run while simulating CD8 depletion and removal of CTL killing at 10 days before interruption of viral replication occurring 94 days after infection, and assessing the slope of viral decay after treatment.

### Sensitivity Testing of Varying Parameter Combinations

The models using either the NPP or PP assumptions for CD4^+^ T lymphocyte generation were re-run using varying parameter combinations, to predict viremia decay after antiretroviral administration with or without CD8 depletion. The varied parameters included the lifetime of an activated CD4^+^ T lymphocyte (4, 7, or 15 days), the basic reproductive ratio for infected cells (8, 10, and 15), the eclipse period for an infected cell (1, 2, and 2.5 days), and the factor for activation/killing rate of CTLs (10^−9^, 5×10^−9^, and 10^−8^), yielding 81 parameter sets (Tables S1 and S2). After running simulations with all combinations of these parameters, some parameter sets were dropped because they resulted in non-containment of viremia in chronic infection (6 sets in the NPP model and 12 in the PP model). The simulations were also run with CD8 depletion as described above. The percent differences in viremia decay rates (slopes of viremia decline after antiretroviral therapy) were calculated as:

100×(slope without depletion–slope with depletion)÷(slope without depletion).

## Results

### Modeling the Biology of T Lymphocyte Generation and SIV Replication *in vivo* According to Biologic Principles and Parameters

The T lymphocyte population is dynamic and heterogeneous, and contains subsets that reflect the lineage of cell development. Maturation and activation are key factors determining the generation of SIV permissive CD4^+^ T lymphocytes and functionally antiviral CD8^+^ T lymphocytes (CTLs), both of which are activated effector-memory T lymphocytes that arise from naïve T lymphocytes. Our previously reported model [28], [29], [40], [41] following this scenario was adapted for the current study. Figures 1 and 2 conceptually summarize our modeling of the generation and fate of infected CD4^+^ T lymphocytes and virus-specific CTLs in SIV-infected macaques.

### Target CD4^+^ T Lymphocyte Generation and Infection, under the NPP Scenario

For the CD4^+^ T lymphocyte compartment (Figure 1), the model begins with the assumption of about 3.5×10^9^ total CD4^+^ T lymphocytes at baseline before infection in a macaque [44], of which about 99% are in a resting state. Resting cells can become activated without proliferation (non-programmed proliferation assumption), with about 4.5% of cells activated at steady state before infection, or die at a rate of 0.1% per day if not activated [45], [46]. Activated cells can survive up to 4 days, after which 95% die and 5% survive as resting memory cells [47]. The macaque is infected with SIV to yield 100 infected activated cells initially. If a cell is infected early enough in its life cycle for the virus to complete its life cycle, virion release by each infected cell commences after an “eclipse period” (intracellular phase of viral replication) of two days, after which the cell produces virions at an exponentially increasing rate [25], [33] for a maximum of another two days in the absence of immune clearance. Infected CD4^+^ T lymphocytes (without immune clearance) can produce up to a maximum of about 20,000 virions (most of which are nonviable), resulting in a basic reproductive rate of each infected cell causing the infection of 10 new cells [48]. Virus production can be interrupted by either predestined cell death (cells infected late in the lifespan) or CTL clearance at a rate proportional to the stage of viral production using a factor previously derived from *in vitro* and *in vivo* data [29]. These parameters are summarized in Table 1.

### SIV-specific CD8^+^ T Lymphocyte (CTL) Generation

For the CD8^+^ T lymphocyte compartment (Figure 2), the generally accepted concept of “programmed proliferation” [36], [37] is followed to model SIV-specific CTLs. At baseline before infection of a macaque, there are about 8.75×10^4^ SIV-specific naïve CTLs based on an assumption of 5 stable epitope-specific responses (ignoring escaped responses), prior measurements of total body CD8^+^ T lymphocytes in macaques, and the biologically observed frequency of precursor CTLs against a single epitope [44], [49]. These precursor (resting) SIV-specific cells either die spontaneously at a rate of 0.1% per day [45], [46] or are activated at a rate dependent on viremia level in the model, after which they are committed to undergo eight divisions [36], [50]. After this division program, 95% of the cells die and the remainder transform to a resting memory state [36], [51]. Resting memory cells either die spontaneously at a rate of 3% per day (based on CTL decay curves in HIV-1-infected persons who received antiretroviral therapy [52], [53], [54]), or get re-activated by antigen into programmed proliferation at a rate that is seven-fold that of naïve cells [47]. Naïve cells require 12 hours for mitosis, while memory cells require 6 hours [55], and naïve cells become mature effector cells after four doublings while memory cells become mature effector cells after a single doubling [55]. These parameters are summarized in Table 2.

### This Model of CTL and SIV Infection Dynamics Accurately Predicts the Course of SIV Infection *in vivo*

**Figure 3:**
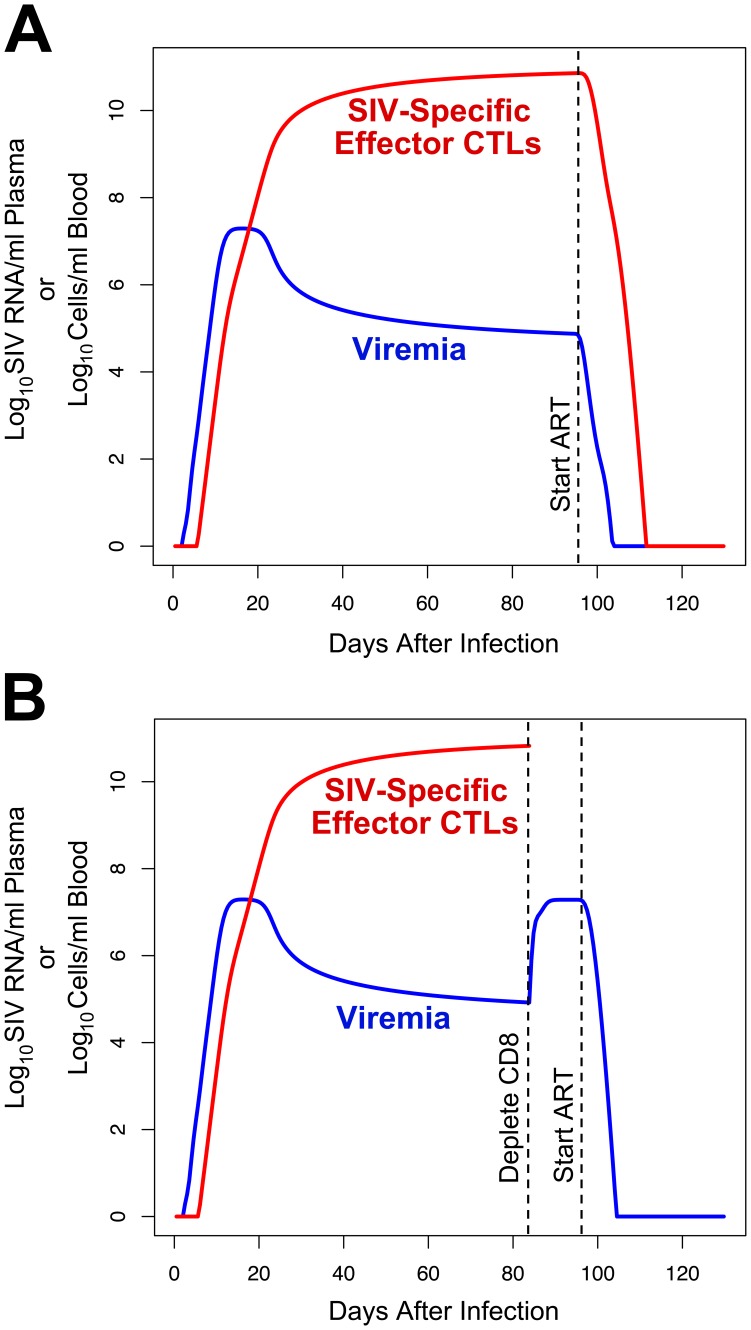
Viremia and CTL dynamics predicted by the model using the non-programmed proliferation (NPP) assumption of CD4^+^ T lymphocyte generation. The levels of viremia and SIV-specific effector CTLs predicted by the model are plotted. A. Curves when antiretroviral therapy is administered at 94 days after infection. B. Curves when CD8 depletion is performed at 84 days after infection, and antiretroviral therapy is administered at 94 days after infection.

Based on the assumptions above, our model predicts a pattern of viremia and SIV-specific CTL expansion that quantitatively reproduces that seen during in acute and chronic infection in the SIV-macaque model (Figure 3). As observed *in vivo* [14], [56], viremia rapidly rises to a peak approximately 10 days after infection and then decreases to a chronic set-point level after the frequency of SIV-specific CTLs rises to a peak about four to eight weeks after infection. Both viremia and CTL levels reach this set-point spontaneously in the model, due to interactions between virus, target CD4^+^ T lymphocytes, and CTLs that reach equilibrium.

Further modeling the administration of antiretroviral therapy after 94 days (assumed to stop SIV replication instantaneously in the model), as tested in the two studies of SIV dynamics by Klatt *et al* [30] and Wong *et al* [31], the predicted viremia decay rate is 1.41 log_10_ units per day (half-life of 0.49 days), resulting in near-clearance by two weeks (Figure 3A). This decay rate is similar to those observed experimentally in macaques in the Klatt and Wong studies, in light of the approximate nature of some biologically measured parameters in our model, and likely pharmacologic/biologic lag and incomplete SIV suppression with antiretroviral drug administration that was not considered in those studies. Klatt *et al* reported viremia half-lives of 0.69 to 1.64 days in four animals (mean 1.1±0.4 days) [30] and Wong *et al* reported half-lives ranging from 1.1 to 4.3 days in three animals (2.3±1.7 days) [31], in agreement with a prior study from Nowak *et al* that reported half-lives of 0.7 to 1.4 days [57]. Overall, these results demonstrate that our model provides a reasonable simulation of the dynamics of viral replication based on the interactions of infected cells and SIV-specific CTLs *in vivo*. In contrast to the simple model utilized in those prior studies, which estimated multiple biologic parameters from the observed set-point viremia level, our model utilized known biological parameters to arrive at the observed steady state.

### The Model Demonstrates that CD8^+^ T Lymphocyte Depletion can Increase Viremia Sharply with a Relatively Small Change in the Lifetime of Infected Cells

The effect of CD8^+^ T lymphocyte depletion on viral dynamics was tested in our model (Figure 3B), to mimic experiments administering anti-CD8 antibody to SIV-infected macaques. The model predicted a rise in viremia to a new plateau approximately 2.4 log_10_ units above the prior steady-state level after about four days, which was similar to that observed in prior CD8 depletion experiments in SIV-infected macaques [11], [12], [13], [14] and the Klatt and Wong studies [30], [31]. Replicating the viral decay experiments in the latter studies, the impact of antiretroviral administration after CD8 depletion was examined. The model predicted a viremia decay rate (slope) after CD8 depletion that increased by 6.8% versus no depletion. Thus our model incorporating cytolytic clearance of infected cells accurately recapitulates the biological results seen by Klatt *et al* and Wong *et al* [30], [31], predicting a relatively small change in viremia decay rate that those studies did not have the statistical power to detect.

### The Finding Predicted by the Model is Robust Across Varying Parameters for Infected Cell Lifetime, Eclipse Period, Viral Reproduction Rate, CTL Activation Rate, and CTL Killing Rate, as Well as the PP CD4^+^ T Lymphocyte Generation Scenario

**Figure 4:**
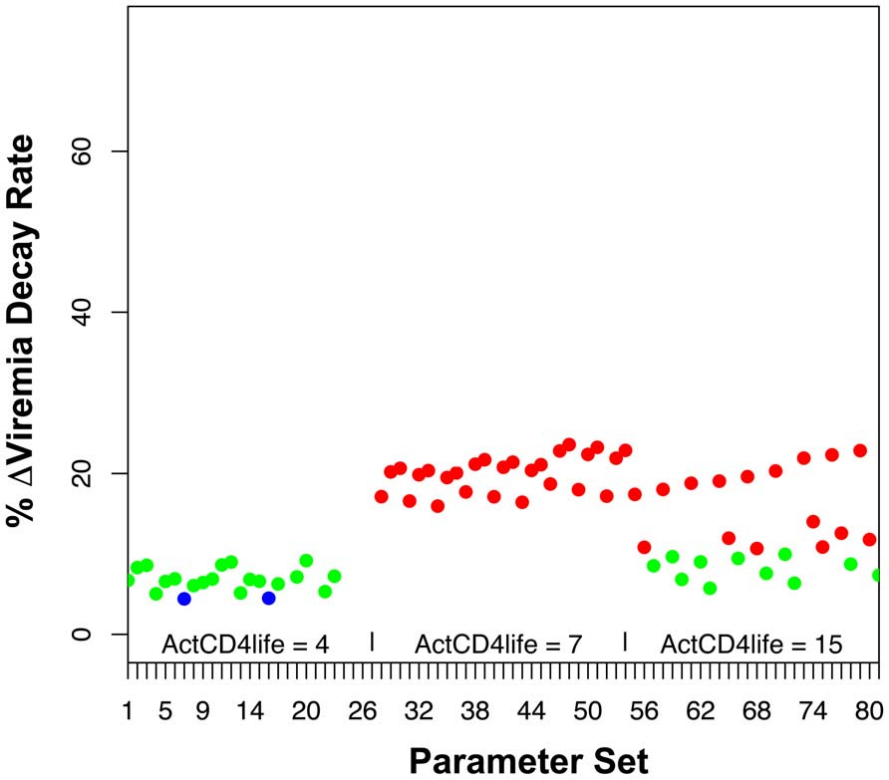
Sensitivity testing of the model using the CD4^+^ T lymphocyte NPP assumption. Key assumed biological parameters in our model were varied in order to test the rigor of the prediction that CD8 depletion causes a relatively small change in rate of viremia decay. The lifetime of an activated CD4^+^ T lymphocyte (ActCD4life) was varied over 4, 7, or 15 days. The basic reproductive rate for infected cells (number of newly infected cells resulting from an infected cell in the absence of immune clearance, *R_0_*) was varied over 8, 10, and 15. The eclipse period for an infected cell was varied over 1, 2, and 2.5 days. The factor for rate of CTL activation and killing of infected cells (α = κ) was varied over 10^−9^, 5×10^−9^, and 10^−8^. The graph plots the predicted % change in decay rate caused by CD8 depletion for each set of the 81 possible combinations of these parameters, varied in order of listing. Predicted decay rates of <5% are shown in blue dots, 5–10% in green dots, and >10% in red dots.

Clearly, the predicted rates of viremia decay after antiretroviral treatment in the absence and presence of CD8 depletion are subject to the parameters and assumptions of our model. Sensitivity testing was performed by varying key input parameters in the model, including the lifetime of activated CD4^+^ T lymphocytes, the reproductive rate of SIV-infected cells, the eclipse period of SIV-infected cells, and the activation/killing rates of CTLs. Each parameter was assigned 3 different values, allowing for 81 possible combinations. The resulting 81 parameter sets (Table S1) were tested in the model to determine viremia decay rates without and with CD8 depletion, and the percentage change induced by CD8 depletion was plotted for each scenario (Figure 4 and Table S1). The predicted changes in viremia decay fell between 4 to 24% (median 12.6%), with generally higher values corresponding to longer activated cell lifespans, as expected due to the longer window for CTL killing and therefore greater impact of killing on lifespan.

**Figure 5:**
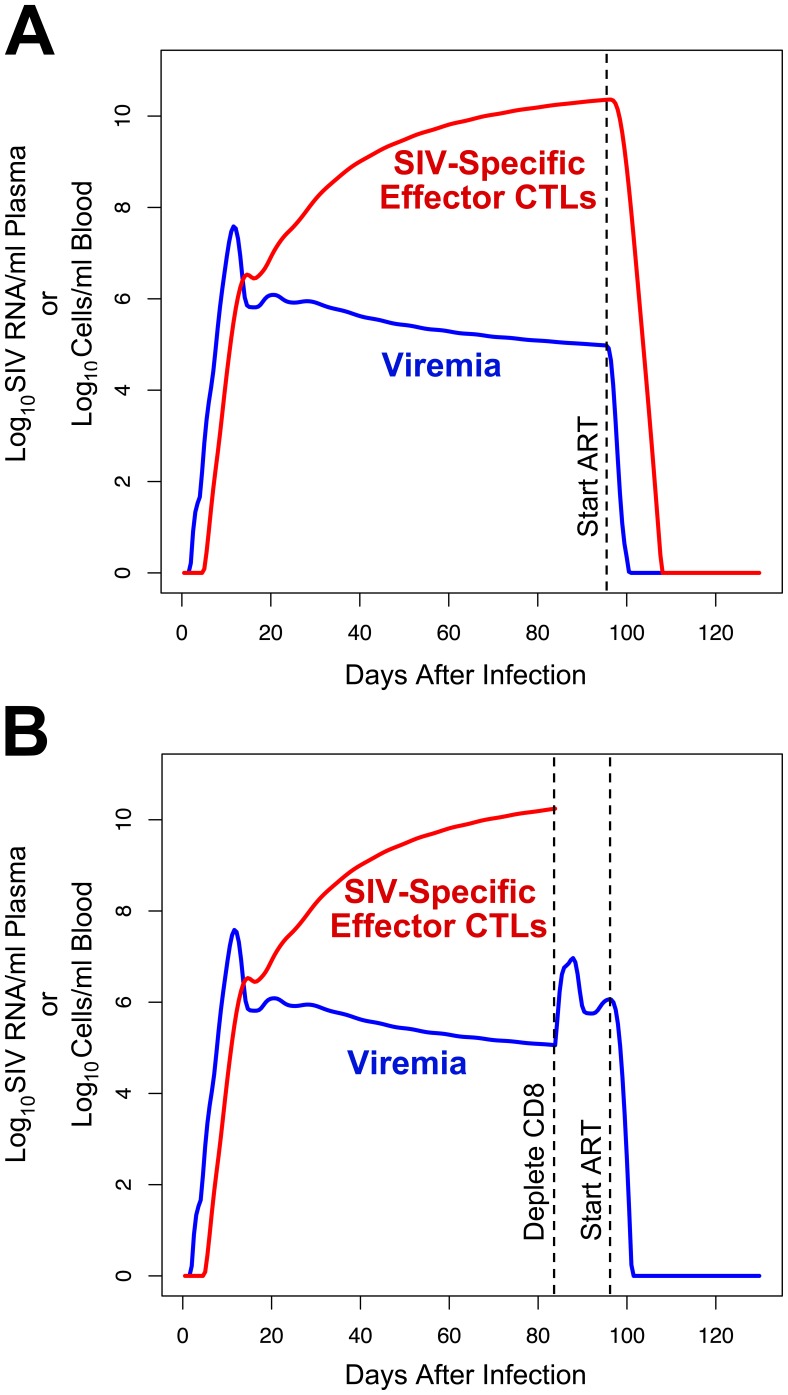
Viremia and CTL dynamics predicted by the model using the programmed proliferation (PP) assumption of CD4^+^ T lymphocyte generation. The levels of viremia and SIV-specific effector CTLs predicted by the model are plotted. A. Curves when antiretroviral therapy is administered at 94 days after infection. B. Curves when CD8 depletion is performed at 84 days after infection, and antiretroviral therapy is administered at 94 days after infection.

**Figure 6:**
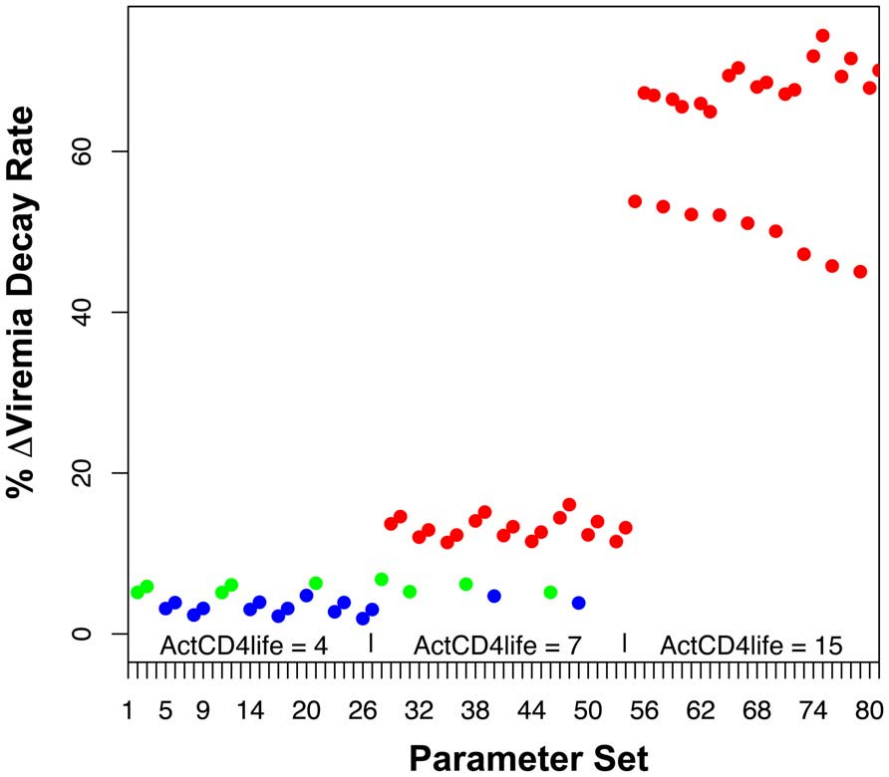
Sensitivity testing of the model using the CD4^+^ T lymphocyte PP assumption. Key assumed biological parameters in our model were varied in order to test the rigor of the prediction that CD8 depletion causes a relatively small change in rate of viremia decay. The lifetime of an activated CD4^+^ T lymphocyte (ActCD4life) was varied over 4, 7, or 15 days. The basic reproductive rate for infected cells (number of newly infected cells resulting from an infected cell in the absence of immune clearance, *R_0_*) was varied over 8, 10, and 15. The eclipse period for an infected cell was varied over 1, 2, and 2.5 days. The factor for rate of CTL activation and killing of infected cells (α = κ) was varied over 10^−8^, 10^−7^, and 5×10^−7^. The graph plots the predicted % change in decay rate caused by CD8 depletion for each set of the 81 possible combinations of these parameters, varied in order of listing. Predicted decay rates of <5% are shown in blue dots, 5–10% in green dots, and >10% in red dots.

A major modeling assumption was the non-programmed proliferation of CD4^+^ T lymphocytes (NPP), given that the process of generating activated CD4^+^ T lymphocytes, most of which are not directly antigen-driven during SIV infection, is poorly understood. An alternative programmed proliferation (PP) scenario [36] therefore was tested. The results demonstrated that this model also produced viremia curves consistent with the pathogenesis of SIV infection in macaques, and also predicted relatively small changes in viremia decay after antiretroviral treatment in the absence or presence of CD8 depletion (Figure 5). Finally, this alternative model was also tested across the same varying parameter assumptions (Table S2). This sensitivity testing also demonstrated relatively small changes in infected cell lifespan after CD8 depletion (Figure 6, and Table S2), ranging from 2 to 74% (median 13.4%), again with the higher values generally corresponding to longer activated cell lifespans. While there was more variability in viremia decay change after CD8 depletion for the PP versus the NPP assumptions, the majority of viable parameter sets (49 of 69) predicted a change of <52.7%, which was the mean change plus one standard deviation in the longitudinally tested animals within the Klatt *et al* study (Table S3). Indeed, 42 of 69 viable parameter sets predicted a change of <20%. These results showed that the findings of the model are robust over reasonable ranges of biological parameters.

## Discussion

It has been over 25 years since the initial observation that CD8^+^ T lymphocytes suppress HIV-1 replication [15]. Soon after that observation, it was noted that most HIV-1-infected persons have vigorous levels of circulating HIV-1-specific, HLA class I-restricted, cytolytic CD8^+^ T lymphocytes (CTLs) [23], [24] and that these cells can mediate potent HLA-restricted antiviral activity [58]. However, debate continued as to whether CTLs mediate antiviral activity via a non-cytolytic and non-epitope-specific manner, or via cytolysis after epitope recognition. The demonstration that HIV-1-specific CTLs suppress viral replication both by cytolysis and production of non-cytolytic soluble factor(s) in an epitope-specific manner [21], [22], [59], but predominately via cytolysis [22], seemed to reconcile these viewpoints in the context of other biological evidence for the role of cytolysis by CTLs.

**Figure 7:**
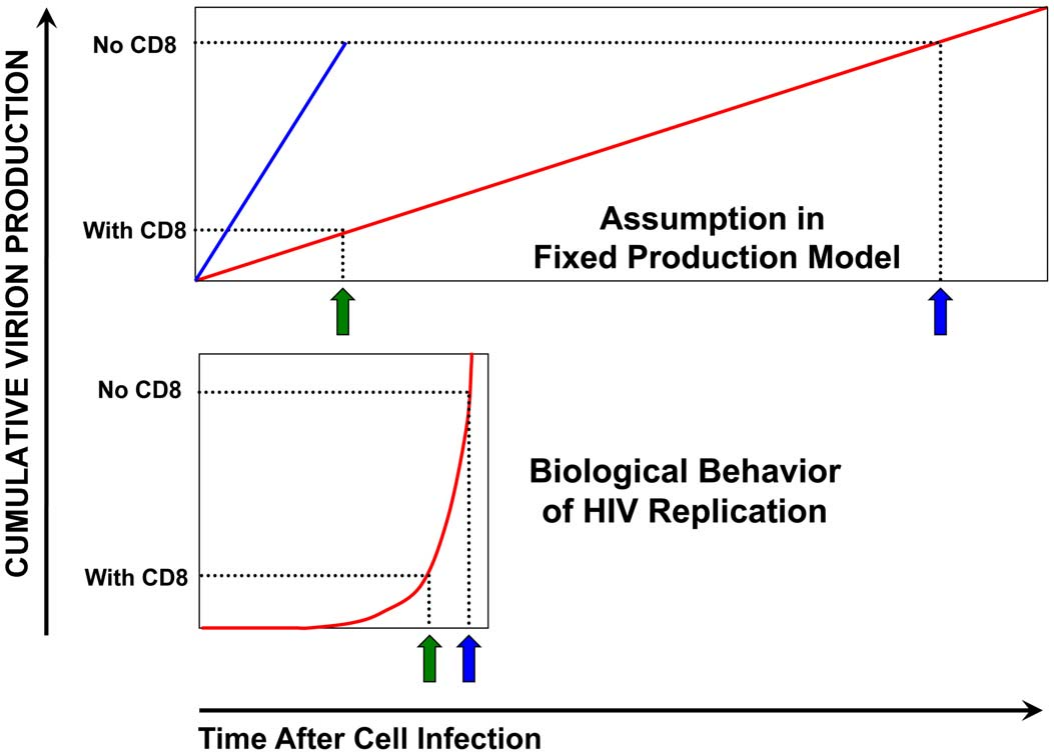
Schematic of different viral replication modeling assumptions. The assumptions and implications of the fixed production model and the model utilized in the current study are compared schematically: Top: The fixed production model assumes that the amount of plasma virus is always in direct proportion to the number of infected cells, and therefore that infected cells produce virus at a constant rate after infection. Based on the observed viremia decay rate after antiretroviral treatment, the average lifetime of an infected cell is calculated (green arrow). After CD8 depletion, the lifetime of an infected cell would have to increase linearly to explain the increased virus (blue arrow) if shortened lifetime were the mechanism of CTL suppression of viral replication. However, Klatt *et al* and Wong *et al* were unable to measure a change in viremia decay rates and therefore assumed that virus production per cell must be increased after removal of CTLs (blue line). Bottom: Our model assumes a non-linear rate of virus production by an infected cell, starting with an eclipse period and then an exponential rise in production, fitting biological observations of viral replication. If CTLs kill the infected cell during the productive phase of infection at baseline (green arrow), loss of CTLs result in a relatively large output in virus production with a small change in the lifetime of the infected cell. Thus the rise in virus production seen after CD8 depletion is consistent with a small change in the lifetime of infected cells, far below the power to detect with a small number of tested macaques.

However, two reports in PLoS Pathogens [30], [31] have suggested that CTLs cannot mediate antiviral activity via cytolysis of infected cells *in vivo* in the SIV-macaque model. Viremia decay rates after antiretroviral treatment were not measurably changed by CD8 depletion, which was interpreted by applying a fixed production model to suggest that the lifetime of infected cells was unchanged after CTL removal. This model [32] assumed that the lifetime of infected CD4^+^ T lymphocytes is directly reflected by the decay rate of plasma viremia due to the very short half-life of virions [60], [61]. Thus because the decay rate of plasma viremia was not detectably changed by the predicted 10- to 20-fold amount required for cytolysis to explain a 10- to 20-fold increase in viremia in their fixed production model, the rise in viremia after CD8 depletion in these studies was attributed to increased virus production by infected cells without lengthened lifespan (Figure 7 top). It was concluded that CTLs do not mediate antiviral activity by killing infected cells.

However, this fixed production modeling assumes, due to the very short survival of free virions, that plasma viremia decay after antiretroviral treatment directly (linearly) reflects the kinetics of infected cell decay. This simple assumption requires two key implicit assumptions about the relationship of plasma viral decay to infected cell lifetime: that infected cells produce virus at a constant rate after infection, and that infected cells have fixed lifespans. Violation of either assumption renders the relationship between rates of changing viremia and changing infected CD4^+^ T lymphocyte levels non-linear. We propose that these assumptions are inconsistent with the biology of SIV/HIV, and demonstrate that a more detailed model incorporating biological principles of virus-CD4^+^ T lymphocyte-CTL interactions supports the role of killing as an antiviral mechanism of CTLs. Our model includes cytolysis as the antiviral mechanism of CTLs, yet accurately recapitulates the viral decay rates observed in the Klatt and Wong studies. Thus, our results provide a counterpoint to their interpretation of the biological data.

In regards to the first point about the fixed production model, the assumption that the decay rate of viremia after antiretroviral therapy is directly related to the lifetime of infected cells requires that infected cells release virions at a constant rate; e.g. a 10-fold increase in infected cell lifetime is required to produce a 10-fold increase in virus output. This assumption immediately leads to contradiction of two biological points. As mentioned in the Introduction, the fixed production model would require activated CD4^+^ T lymphocytes infected by SIV to survive an average of more than 10 days after removal of CTLs, if cytolysis of infected cells maintains the baseline viremia that is more than 10-fold lower (Figure 7 top). Clearly, this is incompatible with the lifespan of activated T lymphocytes even in the absence of infection. Additionally, it is clear that virion production does not occur at a constant rate in infected cells. The life cycle of HIV/SIV includes an intracellular phase of viral replication before infectious virions are released by an infected CD4^+^ T lymphocyte, spanning several steps of replication including uncoating of the viral core, reverse transcription, proviral integration into the host chromosome, transcription and translation of viral genes, and assembly of the proteins that form new virions. Experimentally, significantly detectable release of virions (by p24 ELISA or titer) is not noted until about 48 to 72 hours in acutely infected cells, after which there is an exponential pattern of viral production [25], [33]. Virus production thus is not uniform over the infected cell lifetime, but highly biased towards the end of the infected cell lifetime, and thus a small change in lifetime can mediate a relatively large impact on virion output (Figure 7 bottom). This pattern of viral replication does not require cytolysis to shorten the infected cell lifespan by 10-fold to mediate a 10-fold decrease in virion production, as concluded by Wong *et al* using the fixed production model [31].

The small numbers of animals and high degree of variability of viremia decay rates between animals would not have the power to detect a small change in infected cell lifetime due to CD8 depletion. Wong *et al* [31] cross-sectionally compared viremia decay between groups of 3, 2, and 3 macaques with varying doses of CD8-depleting antibody. Klatt *et al* [30] compared viremia decay in 8 animals without CD8 depletion and 9 animals with CD8 depletion, including 7 animals that were tested longitudinally under both conditions. Even considering only these 7, intra-macaque decay rate comparisons showed great variability, with a mean decay rate increase of 5.5% and standard deviation of 47.2% after CD8 depletion (Table S3).

The other crucial underlying assumption of the Klatt and Wong studies is that infected CD4^+^ T lymphocytes represent a uniform compartment with a fixed lifespan. Biologically, infected cells are a heterogeneous population. The activated memory CD4^+^ T lymphocytes that support HIV/SIV replication are destined to die or revert to resting memory cells within days. Thus, a cell that is infected towards the end of its lifespan is likely to produce fewer virions than one infected at the beginning. Also, recognition of infected cells by CTLs likely depends on the level of antigen presentation, which increases with lifetime of an infected cell; a minority of infected cells may be susceptible to recognition. Finally, it is likely that all infected cells do not encounter virus-specific CTLs at a fixed time after infection, and some cells may survive to complete the viral life cycle. As a whole, CTL recognition is not uniform across the population and may be biased towards the most infected cells, which we include in our model. Cytolysis would shorten the lifetime of this minority selectively, having a minor effect on the average lifetime of the whole population of infected cells. For example, if the overall infected cell population were to have a mean lifespan of 3 days without CTL killing, but CTL killing were to shorten the lifespan of 10% of the population (the most highly infected cells) by 0.5 day, the mean lifespan of the whole population would only be reduced to 2.75 days (0.9×3+0.1×0.5), despite killing of the cells that produce the most virus.

Beyond these caveats to the fixed production model, experimental factors may have contributed to underestimation of the viremia decay rate change after CD8 depletion in the Klatt and Wong studies. Their modeling did not take into account pharmacologic and biologic lag in the reduction of viral replication, which would dilute a difference between CD8 depleted and non-depleted viral decay rates after antiretroviral administration. Another potential contributing factor to underestimating the impact of CTL killing on viral decay is the assumption that CTL activity would remain constant after pharmacologic interruption of SIV replication. There are two reasons that CTL killing activity might decline as viral replication is halted. First, the activation and proliferation of virus-specific CTLs depends on antigenic stimulation, as demonstrated by studies showing declining rates of HIV-1-specific CTLs after antiretroviral treatment of persons [52], [53], [54]. Although Klatt *et al* measured overall CTL frequencies to confirm that SIV-specific CTL levels appeared stable, this would not detect a change in phenotype resulting in reduced killing. Second, as infected cells decrease in frequency, the rate at which they encounter CTLs likely would decrease, also reducing the frequency of killing.

For simplicity, our modeling omitted certain biological concepts. Longer-lived infected cells such as monocytes/macrophages were not included, because their contribution to plasma viremia is quite minor, and they also have been shown to be susceptible to CTL killing [23]. Similarly, the latent reservoir of infected cells was not considered, because it contributes negligibly to plasma viremia [62]. Viral mutational escape from CTL recognition also was not considered, since this factor would decrease the killing efficiency of CTLs (counter our hypothesis), and the stability of CTL responses and viral sequences suggests that this is a negligible factor during the steady state of chronic infection [43].

In conclusion, given the bountiful basic scientific data in animal models regarding the importance of the perforin-granzyme cytolytic pathway in protection from viral infections, and the strong circumstantial evidence in HIV-1 pathogenesis, it seems unreasonable to assume that these cytolytic effector molecules are not present for a key purpose, or that HIV-1 pathogenesis follows entirely different rules compared to other viruses. Our findings underscore the importance of incorporating the complicated interactions of virus with CD4^+^ and CD8^+^ T lymphocytes into models to achieve accurate predictions. The accompanying commentary [63] regarding the two PLoS Pathogens articles disputing CTL killing activity cited former US Secretary of Defense Donald Rumsfeld’s infamous statement about “known and unknown unknowns” to suggest that the cytolytic activity of CTLs should be reclassified in the latter category. Alternatively, we propose that the strength of the basic biologic data about viral replication and T lymphocytes, and the ability to reconcile the experimental findings to a reasonable model that includes CTL killing activity suggest that the role of CTLs as killers should remain a clear “known” despite the “unknowns” that currently prevent complete understanding and complete modeling of the processes involved.

## Supporting Information

Table S1 **Sensitivity study of the model utilizing the non-programmed proliferation (NPP) assumption of CD4^+^ T lymphocyte generation.** Parameter sets were produced by varying individual parameters as indicated. For each parameter set, the model was run to calculate viremia decay slopes after antiretroviral therapy without and with CD8 depletion. The change in slopes between these conditions is indicated for each parameter set. Parameter sets that did not yield containment of viremia in chronic infection were excluded from this analysis (shaded gray). The parameters utilized for Figure 3 are highlighted yellow.
(PDF)

Table S2 **Sensitivity study of the model utilizing the programmed proliferation (PP) assumption of CD4^+^ T lymphocyte generation.** Parameter sets were produced by varying individual parameters as indicated. For each parameter set, the model was run to calculate viremia decay slopes after antiretroviral therapy without and with CD8 depletion. The change in slopes between these conditions is indicated for each parameter set. Parameter sets that did not yield containment of viremia in chronic infection were excluded (shaded gray). The parameters utilized for Figure 5 are highlighted yellow.
(PDF)

Table S3 **Longitudinal changes in viremia decay rates after antiretroviral administration with and without CD8 depletion, as observed in the Klatt** ***et al*** **study.** Raw data from the Klatt *et al* study (Supplemental Table from Klatt NR, Shudo E, Ortiz AM, Engram JC, Paiardini M, et al., 2010, CD8+ lymphocytes control viral replication in SIVmac239-infected rhesus macaques without decreasing the lifespan of productively infected cells, PLoS Pathog 6: e1000747) are analyzed. For each longitudinally tested animal, the change in viremia decay was calculated (bottom boxes). Animals that were not longitudinally tested are shaded in gray.
(PDF)
