# Supplementary material for: Biologically-Directed Modeling Reflects Cytolytic Clearance of SIV-Infected Cells In Vivo in Macaques
Source: PLoS One. 2012 Sep 13;7(9):e44778. doi: 10.1371/journal.pone.0044778 (PMC3441463; doi:10.1371/journal.pone.0044778)
Supplement: Table S1 — Sensitivity study of the model utilizing the non-programmed proliferation (NPP) assumption of CD4+ T lymphocyte generation. Parameter sets were produced by varying individual parameters as indicated. For each parameter set, the model was run to calculate viremia decay slopes after antiretroviral therapy without and with CD8 depletion. The change in slopes between these conditions is indicated for each parameter set. Parameter sets that did not yield containment of viremia in chronic infection were excluded from this analysis (shaded gray). The parameters utilized for Figure 3 are highlighted yellow. (PDF) [file pone.0044778.s001.pdf]

Parameter Set	ActCD4life (Days)	R_0	Eclipse (Days)	α and K (x 10^9)	Viremia Rise After CD8 Depletion	Decay Rate Undepleted	Decay Rate CD8-Depleted	% Δ
1	4	8	1	1	1.85	-1.42	-1.51	6.7
2	4	8	1	5	2.62	-1.40	-1.51	8.3
3	4	8	1	10	2.96	-1.39	-1.51	8.6
4	4	8	2	1	1.60	-1.44	-1.51	5.1
5	4	8	2	5	2.37	-1.42	-1.51	6.6
6	4	8	2	10	2.71	-1.41	-1.51	6.9
7	4	8	2.5	1	1.46	-1.44	-1.51	4.4
8	4	8	2.5	5	2.23	-1.42	-1.51	6.1
9	4	8	2.5	10	2.57	-1.42	-1.51	6.5
10	4	10	1	1	1.85	-1.42	-1.51	6.9
11	4	10	1	5	2.61	-1.39	-1.51	8.7
12	4	10	1	10	2.95	-1.39	-1.51	9.0
13	4	10	2	1	1.61	-1.44	-1.51	5.2
14	4	10	2	5	2.36	-1.41	-1.51	6.8
15	4	10	2	10	2.70	-1.42	-1.51	6.6
16	4	10	2.5	1	1.46	-1.44	-1.51	4.5
17	4	10	2.5	5	2.22	-1.42	-1.51	6.3
18	4	10	2.5	10				
19	4	15	1	1	1.85	-1.42	-1.51	7.2
20	4	15	1	5	2.59	-1.41	-1.51	9.2
21	4	15	1	10				
22	4	15	2	1	1.61	-1.43	-1.51	5.3
23	4	15	2	5	2.36	-1.41	-1.51	7.3
24	4	15	2	10				
25	4	15	2.5	1				
26	4	15	2.5	5				
27	4	15	2.5	10				
28	7	8	1	1	1.93	-0.56	-0.65	17.1
29	7	8	1	5	2.70	-0.54	-0.65	20.2
30	7	8	1	10	3.04	-0.54	-0.65	20.7
31	7	8	2	1	1.82	-0.56	-0.65	16.6
32	7	8	2	5	2.58	-0.54	-0.65	19.9
33	7	8	2	10	2.92	-0.54	-0.65	20.4
34	7	8	2.5	1	1.73	-0.56	-0.65	16.0
35	7	8	2.5	5	2.49	-0.54	-0.65	19.5
36	7	8	2.5	10	2.83	-0.54	-0.65	20.1
37	7	10	1	1	1.94	-0.55	-0.65	17.7
38	7	10	1	5	2.69	-0.54	-0.65	21.2
39	7	10	1	10	3.03	-0.54	-0.65	21.7
40	7	10	2	1	1.82	-0.55	-0.65	17.1
41	7	10	2	5	2.57	-0.54	-0.65	20.8
42	7	10	2	10	2.92	-0.54	-0.65	21.4
43	7	10	2.5	1	1.73	-0.56	-0.65	16.4
44	7	10	2.5	5	2.48	-0.54	-0.65	20.4
45	7	10	2.5	10	2.82	-0.53	-0.65	21.1
46	7	15	1	1	1.94	-0.55	-0.65	18.7
47	7	15	1	5	2.68	-0.53	-0.65	22.8
48	7	15	1	10	3.02	-0.53	-0.65	23.6
49	7	15	2	1	1.83	-0.55	-0.65	18.0
50	7	15	2	5	2.57	-0.53	-0.65	22.4
51	7	15	2	10	2.90	-0.53	-0.65	23.3
52	7	15	2.5	1	1.74	-0.55	-0.65	17.2
53	7	15	2.5	5	2.48	-0.53	-0.65	21.9
54	7	15	2.5	10	2.81	-0.53	-0.65	22.9
55	15	8	1	1	1.67	-0.12	-0.14	17.4
56	15	8	1	5	2.29	-0.12	-0.13	10.8
57	15	8	1	10	2.57	-0.12	-0.13	8.5
58	15	8	2	1	1.65	-0.12	-0.14	18.0
59	15	8	2	5	2.25	-0.12	-0.13	9.7
60	15	8	2	10	2.51	-0.12	-0.13	6.9
61	15	8	2.5	1	1.63	-0.11	-0.14	18.8
62	15	8	2.5	5	2.21	-0.12	-0.13	9.0
63	15	8	2.5	10	2.46	-0.12	-0.13	5.8
64	15	10	1	1	1.69	-0.11	-0.14	19.1
65	15	10	1	5	2.32	-0.12	-0.13	12.0
66	15	10	1	10	2.60	-0.12	-0.13	9.5
67	15	10	2	1	1.67	-0.11	-0.14	19.6
68	15	10	2	5	2.28	-0.12	-0.13	10.7
69	15	10	2	10	2.55	-0.12	-0.13	7.6
70	15	10	2.5	1	1.65	-0.11	-0.14	20.3
71	15	10	2.5	5	2.24	-0.12	-0.13	10.0
72	15	10	2.5	10	2.50	-0.12	-0.13	6.4
73	15	15	1	1	1.71	-0.11	-0.14	21.9
74	15	15	1	5	2.35	-0.12	-0.14	14.0
75	15	15	1	10	2.64	-0.12	-0.13	10.9
76	15	15	2	1	1.70	-0.11	-0.14	22.3
77	15	15	2	5	2.31	-0.12	-0.13	12.6
78	15	15	2	10	2.60	-0.12	-0.13	8.8
79	15	15	2.5	1	1.68	-0.11	-0.14	22.9
80	15	15	2.5	5	2.28	-0.12	-0.13	11.8
81	15	15	2.5	10	2.55	-0.12	-0.13	7.4
