# Supplementary material for: Biologically-Directed Modeling Reflects Cytolytic Clearance of SIV-Infected Cells In Vivo in Macaques
Source: PLoS One. 2012 Sep 13;7(9):e44778. doi: 10.1371/journal.pone.0044778 (PMC3441463; doi:10.1371/journal.pone.0044778)
Supplement: Table S3 — Longitudinal changes in viremia decay rates after antiretroviral administration with and without CD8 depletion, as observed in the Klatt et al study. Raw data from the Klatt et al study (Supplemental Table from Klatt NR, Shudo E, Ortiz AM, Engram JC, Paiardini M, et al., 2010, CD8+ lymphocytes control viral replication in SIVmac239-infected rhesus macaques without decreasing the lifespan of productively infected cells, PLoS Pathog 6: e1000747) are analyzed. For each longitudinally tested animal, the change in viremia decay was calculated (bottom boxes). Animals that were not longitudinally tested are shaded in gray. (PDF) [file pone.0044778.s003.pdf]

Early Phase

**CD8+ Lymphocyte-Depleted**							**Non-Depleted Controls**						
		95% Confidence Interval*			95% Confidence Interval*				95% Confidence Interval*			95% Confidence Interval*	
**Animal**	δ	**Lower**	**Upper**	μ	**Lower**	**Upper**	**Animal**	δ	**Lower**	**Upper**	μ	**Lower**	**Upper**
RRf6	1.15	1.00	1.34	0.40	0.26	0.49	RMm6	0.73	0.67	0.81			
RAj7	0.60	0.51	0.71				RSq8	1.10	0.97	1.28	0.05	0.00	0.09
RLi6	1.22	1.03	1.46	0.22	0.13	0.30	RUe7	1.64	1.49	1.78	0.13	0.10	0.15
RPp6	0.74	0.45	1.50				RWf7	0.69	0.54	0.90	0.18	0.04	0.26
RZl5	1.22	1.03	1.43	0.23	0.18	0.28	XHB	1.07	0.91	1.23	0.13	0.07	0.17
Mean	*0.99*			*0.28*			*Mean*	*1.05*			*0.12*		
sd	*0.29*			*0.10*			*sd*	*0.38*			*0.05*		

Late Phase

**CD8+ Lymphocyte-Depleted**							**Non-Depleted Controls**						
		95% Confidence Interval*			95% Confidence Interval*				95% Confidence Interval*			95% Confidence Interval*	
**Animal**	δ	**Lower**	**Upper**	μ	**Lower**	**Upper**	**Animal**	δ	**Lower**	**Upper**	μ	**Lower**	**Upper**
RSq8	1.99	1.56	2.62	0.20	0.17	0.23	RRf6	1.51	0.98	3.39	0.40	0.00	0.57
RUe7	0.92	0.53	2.10	0.03	0.00	0.19	RAj7	1.16	0.83	1.70	0.10	0.01	0.17
RWf7	0.92	0.52	1.48	0.12	0.06	0.16	RLi6	0.92	0.70	1.17	0.16	0.13	0.20
XHB	1.15	0.90	1.57	0.10	0.02	0.18							
Mean	*1.25*			*0.11*			*Mean*	*1.20*			*0.22*		
sd	*0.51*			*0.07*			*sd*	*0.30*			*0.16*		

***Confidence intervals computed from 500 bootstrap replicates**

Depletion:	δ N	δ Y	% Δ
RAj7	1.16	0.60	-48.3%
RLi6	0.92	1.22	32.6%
RMm6	0.73		
RPp6		0.74	
RRf6	1.51	1.15	-23.8%
RSq8	1.10	1.99	80.9%
RUe7	1.64	0.92	-43.9%
RWf7	0.69	0.92	33.3%
RZl5		1.22	
XHB	1.07	1.15	7.5%

EARLY	mean	-13.2%
	s.d.	41.5%
LATE	mean	19.5%
	s.d.	52.0%
**ALL**	**mean**	**5.5%**
	**s.d.**	**47.2%**
